# What Do Older Adults Think About Virtual Reality: Findings From a Preference Study

**DOI:** 10.1093/geroni/igaf122.1639

**Published:** 2025-12-31

**Authors:** Sara Czaja, Walter Boot, Neil Charness, Shenghao Zang, George Mois, Pallabi Bhowmick, Wendy Rogers

**Affiliations:** Weill Cornell Medicine, New York, New York, United States; Weill Cornell Medicine, New York, New York, United States; Weill Cornell Medicine, New York, New York, United States; Weill Cornell Medicine, New York, New York, United States; University of Georgia, Athens, Georgia, United States; University of Illinois Urbana-Champaign, Champaign, Illinois, United States; University of Illinois Urbana-Champaign, Champaign, Illinois, United States

## Abstract

Social and cognitive engagement are critical elements of successful aging. Yet many older adults, especially those who live alone or have a chronic condition or disability, lack social connectivity, experience loneliness, and lack opportunities for cognitive engagement. Virtual Reality (VR) applications can play a key role in enhancing social and cognitive engagement and decreasing loneliness among older adults. VR provides an immersive and interactive experience, which enables users to feel “present” in an environment, and have the ability to interact solely or with others in a virtual space or activity (e.g., museum, gardening). The presentation will focus on data from a cross-site study that examined perceptions of value, enjoyments and usability (comfort, safety) of VR applications as well as preferences for VR apps among a sample of 48 of community dwelling older adults aged 65 years and older. Sixteen pre-selected apps that focused on social, cognitive and productive engagement were examined. Each participants received a brief training on the use of VR and were exposed to eight apps either interactively or in a video format (apps were counter-balanced across participants). They rated each app after exposure, completed the Attitudes Toward VR Scale, A Virtual Reality Activity Inventory, and an Opinion Interview. The findings indicated that the older adults rated the experience of VR as valuable and highly enjoyable. There were also low ratings of discomfort and high ratings of usability. Most participants indicated that they would like to engage with VR in the future.

